# Replication of the association of the *COG6* gene with juvenile idiopathic arthritis (JIA)

**DOI:** 10.1186/1546-0096-9-S1-P281

**Published:** 2011-09-14

**Authors:** A Hinks, J Cobb, P Martin, E Flynn, S Eyre, J Packham, A Barton, J Worthington, W Thomson

**Affiliations:** 1 Arthritis Research UK Epidemiology Unit, Manchester Academic Health Science Centre (MAHSC), Stopford Building, University of Manchester, Manchester, UK; 2 Haywood Hospital, University Hospital of North Staffordshire, Stoke on Trent, Staffordshire, UK

## Background

A large US JIA case control study investigating SNPs previously implicated in autoimmune diseases recently identified three novel JIA loci, protein tyrosine phosphatase, non-receptor type 2 (*PTPN2*), conserved oligomeric golgi complex component 6 (*COG6*) and angiopoietin 1 (*ANGPT1*). We have previously shown association of SNPs in the *PTPN2* gene in our UK JIA cohort.

## Aim

To test whether the two additional loci also confer susceptibility to JIA in a UK cohort.

## Methods

Two SNPs, rs7993214, near the *COG6* gene and rs1010824, near the *ANGPT1* gene, were genotyped in UK JIA cases (n=817) and the genotype and allele frequency data compared with those for healthy controls (n=5196) extracted from the Wellcome Trust case control consortium 2 (WTCCC2) European Genome-phenome Archive (EGA). The Cochrane-Armitage trend test implemented in PLINK was performed and allelic odds ratios (ORs) and their 95% confidence intervals (CIs) calculated.

## Results

This study had >90% power to detect an effect equivalent to that identified for these loci in the original study. No association was found for the SNP in *ANGPT1* (ptrend = 0.85). The SNP near the *COG6* gene, rs7993214, was significantly associated with JIA overall (ptrend = 0.03 OR 0.9 95% CI 0.81-0.99). Analysis of only the oligoarthritis and RF negative polyarthritis subtypes, the subtypes investigated in the US study, revealed a trend towards association (ptrend = 0.06 OR 0.9 95% CI 0.8-1.01). Our study suggests however that this SNP maybe associated in other JIA subtypes, indeed despite comparatively small numbers (n=123) the association was also significant in the systemic onset subtype (p=0.03). Meta-analysis of this study with the original study provided genome wide significant association of this SNP with JIA (combined p-value=8.5 x 10^-7^ OR 0.84 95% CI 0.78-0.9) with no evidence of heterogeneity between the two studies.

## Conclusions

We present replication of the association of a SNP near the *COG6* gene and JIA. Meta-analysis of this study with the original US study provided genome wide significance for this locus in oligoarticular JIA and RF negative polyarthritis JIA. The SNP lies within an intergenic region between the *COG6* and *FOXO1* genes. COG6 is a component of the conserved oligomeric golgi complex, which plays a role in glycoprotein modification and intracellular transport. FOXO1 belongs to the forkhead family of transcription factors and may be important in regulatory T cell development and function. Further genetic analysis will be required to identify the causal SNP and gene important in JIA susceptibility.

